# Supplementary material for: TRAIL promotes the polarization of human macrophages toward a proinflammatory M1 phenotype and is associated with increased survival in cancer patients with high tumor macrophage content
Source: Front Immunol. 2023 Sep 21;14:1209249. doi: 10.3389/fimmu.2023.1209249 (PMC10551148; doi:10.3389/fimmu.2023.1209249)
Supplement: Supplementary file 1 [file DataSheet_1.pdf]

Supplementary Data

Supplementary Tables

Table S1. FDR and fold change values of differentially expressed M1 and M2 markers in primary human M0, M1, M2a, and M2c macrophages upon TRAIL treatment. According to RNA sequencing analysis, TRAIL-induced differentially expressed M1 and M2 markers in (A) M0, (B) M1, (C) M2a, and (D) M2c macrophage subtypes were determined (n=3-4). FDR values and fold changes of markers were depicted as tables. **(Supplement of Figure 2)**

A

M0 Macrophages

M1 Genes	LogFC	LogCPM	LR	P-Value	FDR	Fold Change
CCL15	2,907937	-1,274537	38,70564	4,93E-10	9,62E-08	7,505439771
CXCL11	2,734056	0,0373049	68,3073	1,4E-16	9,47E-14	6,65323491
CXCL10	2,608033	2,530077	29,67207	5,12E-08	5,89E-06	6,096720046
IFI44L	2,087466	3,8332614	64,71575	8,65E-16	4,82E-13	4,250008526
CXCL1	1,874095	2,9751543	71,44037	2,86E-17	2,39E-14	3,665715319
IL12B	1,814687	-2,129927	12,88084	0,000332	0,01117	3,517831879
IDO1	1,682171	1,9116785	46,62585	8,59E-12	2,45E-09	3,209104641
ACOD1	1,669245	-0,286877	29,94927	4,43E-08	5,3E-06	3,18048193
CD38	1,374008	2,7878062	68,28464	1,42E-16	9,47E-14	2,591896568
IL1B	0,747764	3,5410055	4,33445	0,037348	0,248974	1,679188778
**M2 Genes**	**LogFC**	**LogCPM**	**LR**	**P-Value**	**FDR**	**Fold Change**
SELENOP	-1,59326	4,6101703	24,93365	5,93E-07	5,51E-05	-3,017304424
HGF	-1,49268	4,2914729	29,08246	6,94E-08	7,73E-06	-2,814112767
F13A1	-1,42713	-0,291227	9,979399	0,001583	0,035955	-2,68911441
FAXDC2	-1,27713	4,1928475	12,88344	0,000332	0,01117	-2,423565133
MS4A6A	-1,16267	5,6874632	17,60946	2,71E-05	0,001531	-2,238707242
TMEM37	-1,09938	4,2562729	42,63845	6,59E-11	1,49E-08	-2,142629269
HTR2B	-0,47738	1,9504305	3,053927	0,080542	0,363538	-1,3922159
PRR5L	-0,26564	2,9720773	3,074031	0,079552	0,360884	-1,202171658
ANGPTL4	-0,14318	-0,144882	0,212906	0,644499	0,869164	-1,104333412
HPGD	-0,139	3,5830761	1,329761	0,248848	0,600809	-1,101141603

B

M1 Macrophages

M1 Genes	LogFC	LogCPM	LR	P-Value	FDR	Fold Change
CXCL10	0,828947	11,228708	2,327726	0,127087	0,965829	1,77638816
CXCL11	0,822665	9,8807491	1,64701	0,199366	0,965829	1,768670619
IDO1	0,68372	10,636941	2,06094	0,151117	0,965829	1,606276149
CD38	0,449123	8,7203642	3,482029	0,062039	0,965829	1,365210544
IFI44L	0,385988	8,7063798	3,474881	0,062307	0,965829	1,306754076
ACOD1	0,155129	10,641016	0,993177	0,318967	0,965829	1,113521407
CCL15	0,0036	4,2821061	0,000529	0,981656	1	1,002498528
CXCL1	-0,15699	5,8439745	0,650235	0,420029	0,965829	-1,114960656
IL12B	-0,35411	9,9254864	0,925849	0,335944	0,965829	-1,278193567
IL1B	-0,44167	11,140957	1,828917	0,176256	0,965829	-1,358174659
**M2 Genes**	**LogFC**	**LogCPM**	**LR**	**P-Value**	**FDR**	**Fold Change**
TMEM37	-1,32049	-1,105387	3,747579	0,052884	0,965829	-2,49750808
ANGPTL4	-0,98658	3,1596824	3,76906	0,052209	0,965829	-1,981485499
HPGD	-0,48634	2,1677845	1,002175	0,316785	0,965829	-1,400887258
FAXDC2	-0,29119	0,6192352	0,637408	0,424651	0,965829	-1,22365007
MS4A6A	-0,23175	3,8016707	1,266893	0,26035	0,965829	-1,174258843
HTR2B	-0,08973	-1,410394	0,026217	0,871373	0,995379	-1,064168801
SELENOP	-0,05307	2,1319047	0,053723	0,816707	0,989967	-1,037471183
F13A1	0,194691	-1,682	0,111502	0,73844	0,982077	1,144478827
PRR5L	0,137365	0,3953011	0,14339	0,704934	0,981564	1,099894234
HGF	0,119124	0,7295354	0,178002	0,673096	0,977811	1,086075062

C

M2a Macrophages

M1 Genes	LogFC	LogCPM	LR	P-Value	FDR	Fold Change
CXCL10	2,846259	0,2848345	7,784432	0,00527	0,041815	7,191330785
IL12B	2,787451	-3,782405	7,530861	0,006065	0,046259	6,904087519
IFI44L	1,980685	2,1138425	203,2681	4,04E-46	4,51E-43	3,946803029
ACOD1	1,923619	-2,275701	17,71986	2,56E-05	0,000531	3,793734083
CXCL1	1,691321	-0,956927	32,14145	1,43E-08	6,29E-07	3,229522316
IL1B	1,597582	1,9860465	8,996606	0,002705	0,025411	3,026355753
CXCL11	1,581527	-1,267195	18,66776	1,56E-05	0,000347	2,992865402
IDO1	1,502411	-0,291499	38,79287	4,71E-10	2,79E-08	2,833158513
CCL15	1,278511	-0,849975	17,46876	2,92E-05	0,000595	2,42588452
CD38	1,152447	1,5231751	71,05027	3,48E-17	5,42E-15	2,222906326
**M2 Genes**	**LogFC**	**LogCPM**	**LR**	**P-Value**	**FDR**	**Fold Change**
TMEM37	-0,94794	2,691378	68,89079	1,04E-16	1,55E-14	-1,929122046
HTR2B	-0,91003	-0,673713	10,71438	0,001063	0,012124	-1,87908426
FAXDC2	-0,60759	3,2927398	45,33441	1,66E-11	1,29E-09	-1,523713262
F13A1	-0,53332	6,2318302	91,1175	1,35E-21	3,12E-19	-1,447258189
SELENOP	-0,4562	3,1122743	29,00386	7,22E-08	2,84E-06	-1,37192424
HGF	-0,44038	1,9876509	13,89605	0,000193	0,00303	-1,356964674
MS4A6A	-0,42668	6,903627	131,6616	1,77E-30	9,89E-28	-1,344141155
PRR5L	-0,18533	2,5553107	3,744915	0,052968	0,214471	-1,13707474
ANGPTL4	-0,14291	1,9210136	0,148984	0,699508	0,877811	-1,1041264
HPGD	-0,05864	3,2223639	0,455496	0,499737	0,757905	-1,041484947

D

M2c Macrophages

M1 Genes	LogFC	LogCPM	LR	P-Value	FDR	Fold change
CXCL11	3,056515	-1,714187	45,36932	1,63E-11	1,69E-09	8,319604503
CXCL10	2,560395	0,582034	27,54149	1,54E-07	6,83E-06	5,898690359
ACOD1	2,445728	0,583153	119,1987	9,47E-28	7,46E-25	5,448004036
IDO1	2,019917	1,462388	103,7551	2,29E-24	1,39E-21	4,05560548
IFI44L	1,839096	1,60344	164,6231	1,11E-37	1,85E-34	3,577857388
IL1B	1,615173	3,478197	15,30247	9,16E-05	0,001987	3,06348235
CCL15	1,545454	-1,560201	18,19281	2E-05	0,000533	2,918958104
CXCL1	1,40164	1,231705	49,70747	1,78E-12	2,25E-10	2,642018353
CD38	1,276619	3,115441	171,013	4,45E-39	8,5E-36	2,422705544
IL12B	1,577633	-3,766018	2,903811	0,08837	0,34795	2,984796635
**M2 Genes**	**LogFC**	**LogCPM**	**LR**	**P-Value**	**FDR**	**Fold change**
F13A1	-1,30197	1,745999	68,73848	1,12E-16	2,64E-14	-2,465646274
SELENOP	-0,84189	3,725716	25,92361	3,55E-07	1,46E-05	-1,792395723
HGF	-0,74409	3,939544	15,8374	6,9E-05	0,00157	-1,674920481
MS4A6A	-0,72657	6,165134	184,6165	4,76E-42	1,27E-38	-1,65470461
TMEM37	-0,71433	4,67419	130,9267	2,57E-30	3,12E-27	-1,640718384
PRR5L	-0,65375	3,796947	39,72088	2,93E-10	2,39E-08	-1,573251528
ANGPTL4	-0,65299	0,255135	9,224338	0,002388	0,028861	-1,572421318
HPGD	-0,62477	4,922649	37,4014	9,62E-10	6,95E-08	-1,541965352
FAXDC2	-0,36267	3,015827	12,19579	0,000479	0,007843	-1,285801938
HTR2B	0,141614	0,523952	0,626865	0,428508	0,753493	1,103138722

Supplementary Figures

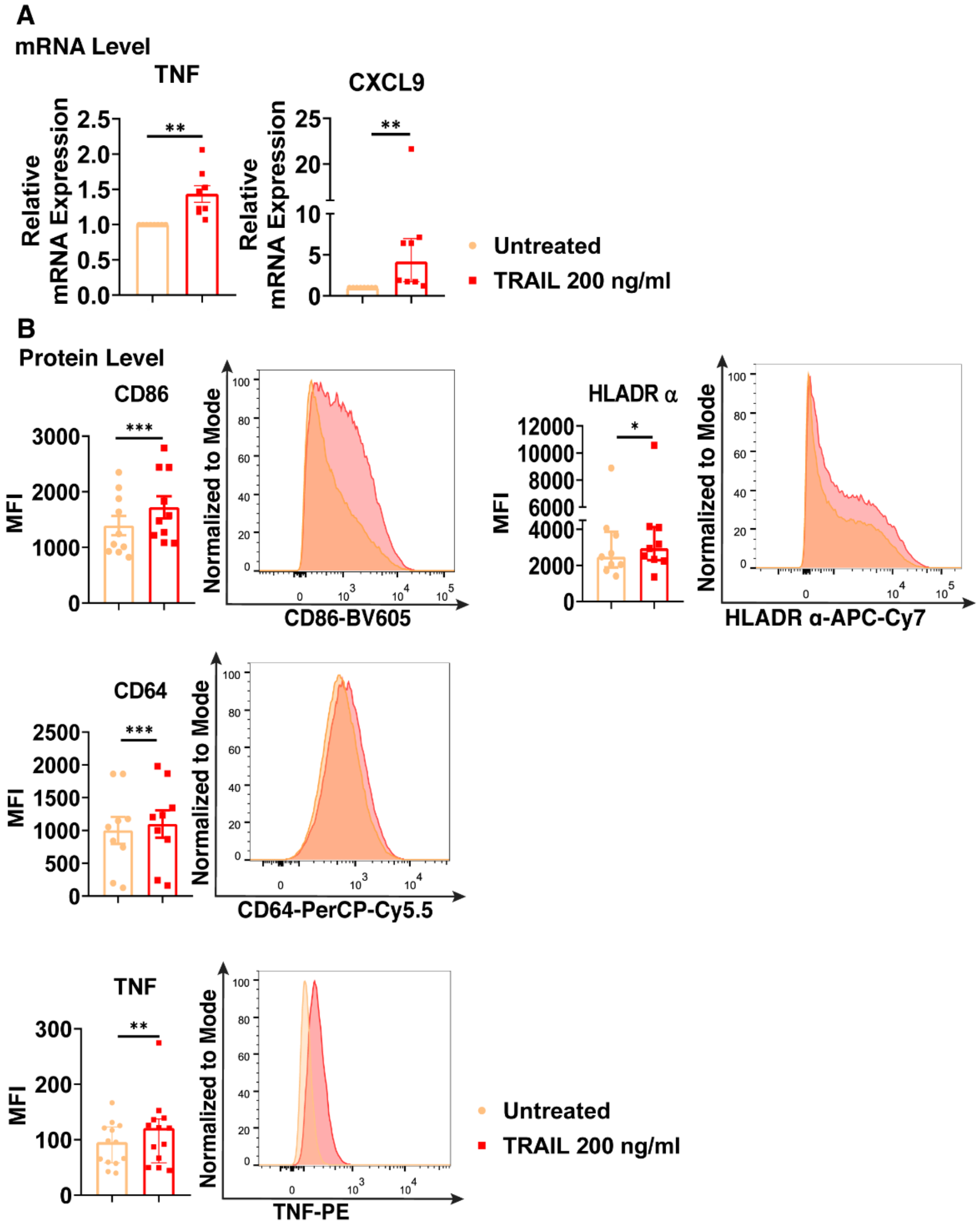

Figure S1. TRAIL increases the expression of classical M1 markers in primary human M0 macrophages at mRNA and protein levels. (A) M0 macrophages were stimulated with 200 ng/ml TRAIL for 8 hours. Control groups were left unstimulated. Expression of TNF and CXCL9 was analyzed by qPCR. (B) M0 macrophages were stimulated with TRAIL for 18 hours (CD86, HLA-DR alpha, CD64) or 6 hours (TNF). Control groups were left unstimulated. Expression of CD86, HLA-DR alpha, CD64 and TNF was analyzed by flow cytometry and representative plots are included. Data shown are mean ± SEM or median with interquartile range pooled from three or more independent experiments [(A) n=8, (B) n=9-13]. Statistical analyses were performed with a two-tailed paired Student's t-test or Wilcoxon matched-pairs signed-rank test between untreated and TRAIL-treated macrophages, *P<0.05, **P<0.01, ***P<0.001.

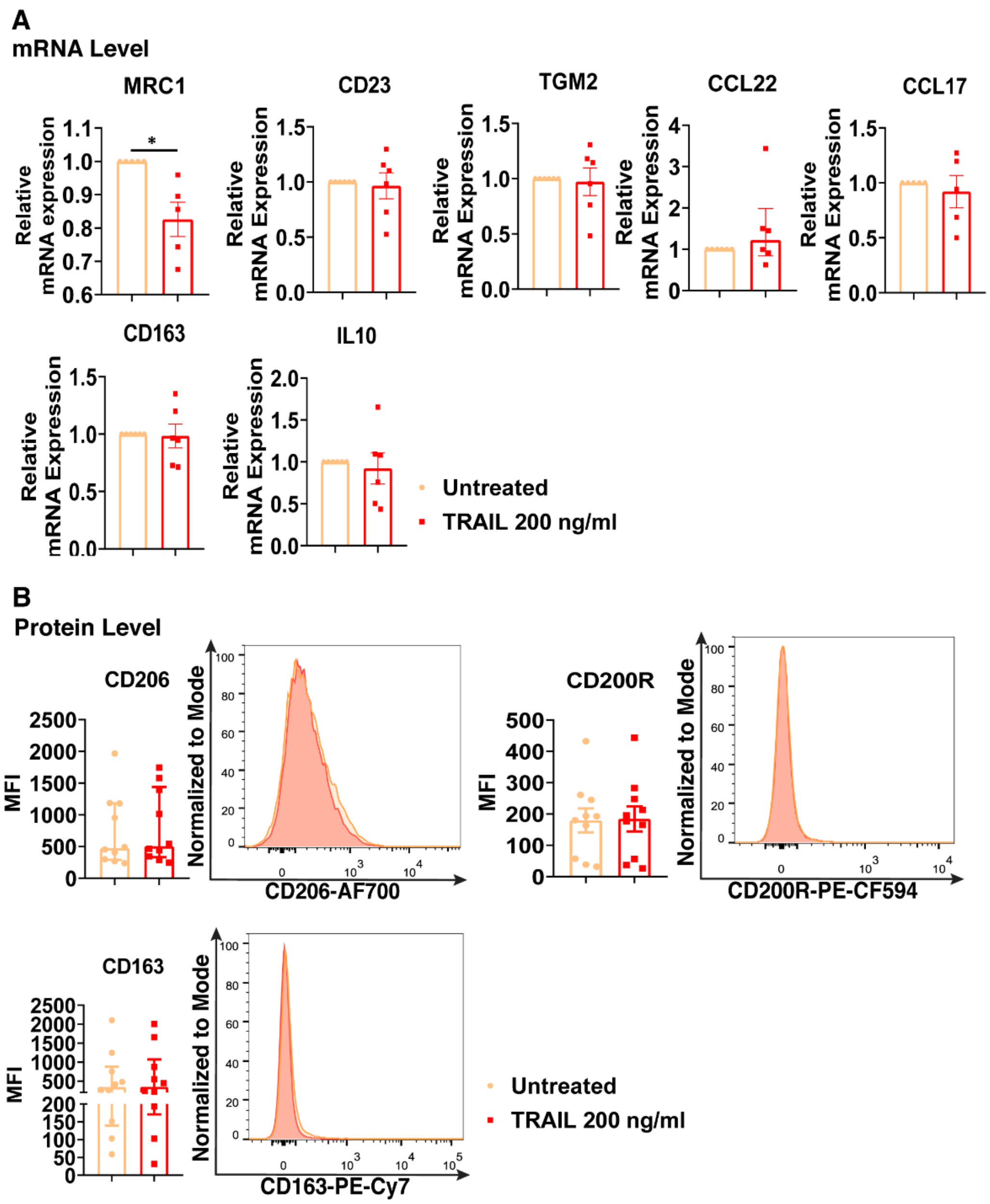

Figure S2. TRAIL does not affect the expression classical M2 markers in primary human M0 macrophages at mRNA and protein levels. (A) M0 macrophages were stimulated with 200 ng/ml TRAIL for 8 hours. Control groups were left unstimulated. Expression of M2 markers was analyzed by qPCR. (B) M0 macrophages were stimulated with TRAIL for 18 hours. Control groups were left unstimulated. Expression of CD206, CD200R, and CD163 was analyzed by flow cytometry, and representative plots are included. Data shown are mean ± SEM or median with interquartile range pooled from three or more independent experiments [(A) n=5-6, (B) n=10]. Statistical analyses were performed with a two-tailed paired Student's t-test or Wilcoxon matched-pairs signed-rank test between untreated and TRAIL-treated macrophages, *P<0.05.

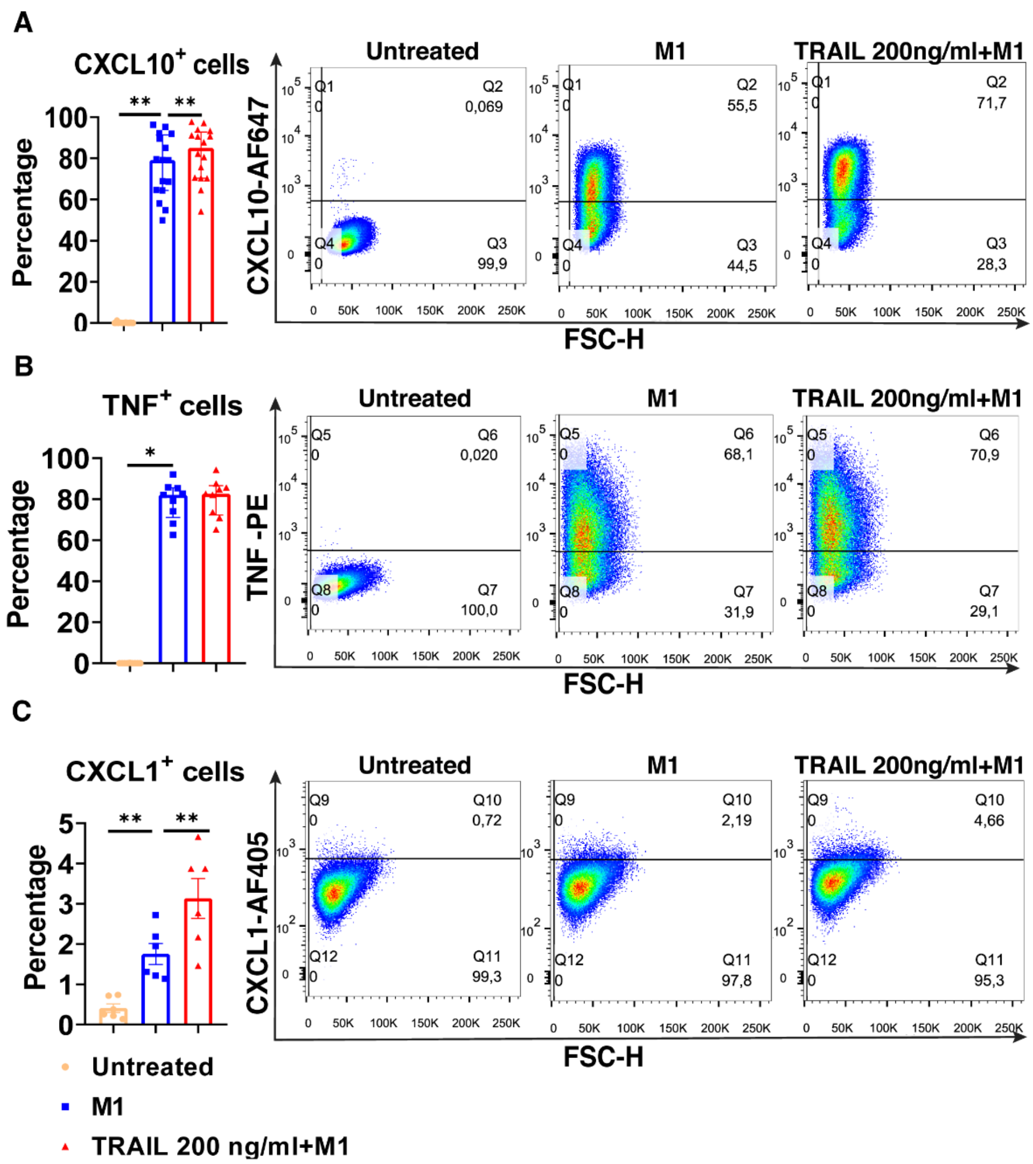

Figure S3. TRAIL increases the percentage of M1 chemokine producing cell populations in primary human M1 macrophages at the protein level. Macrophages were pre-stimulated with 200 ng/ml TRAIL for 2 hours and then polarized into M1 with 100 ng/ml LPS and 20 ng/ml IFNγ for 6 hours. Control groups were left unstimulated or stimulated with only M1 polarization factors for 6 hours. (A-C) The percentage of "CXCL10, TNF, and CXCL1" producing cell populations was analyzed by flow cytometry, and representative plots are shown. Data shown are mean ± SEM or median with interquartile range pooled from two or more independent experiments [(A) n=16, (B) n=9, (C) n=6]. Statistical analyses were performed with a One-way ANOVA with Sidak's multiple comparisons post-hoc test, or Friedman with Dunn's multiple comparisons post-hoc test between untreated and M1 macrophages, M1 and TRAIL-treated M1 macrophages, **P<0.01, ***P<0.001. **(Supplement of Figure 7)**

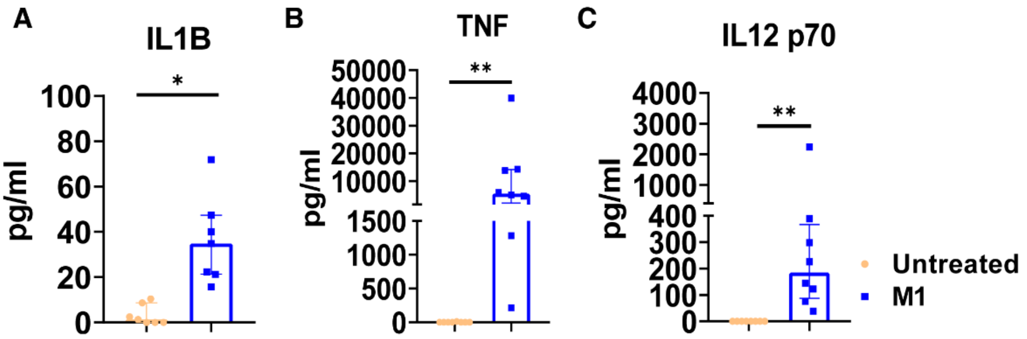

Figure S4. M1 macrophages increases the production of IL-1β, IL-12 and TNF cytokines at the protein level. Cytokine release of the macrophages, used in the ELISA analysis of Figure 7B, at the basal level and after M1 polarization is shown. Macrophages were polarized into M1 with 100 ng/ml LPS and 20 ng/ml IFNγ for 12 hours. Control groups were left unstimulated. Production of cytokines was analyzed by ELISA. Data shown are median with interquartile range pooled from three or more independent experiments [(A) n=7, (B) n=8, (C) n=8]. Statistical analyses were performed with a Wilcoxon matched-pairs signed-rank test between untreated and M1 macrophages, *P<0.05, **P<0.01 **(Supplement of Figure 7).**

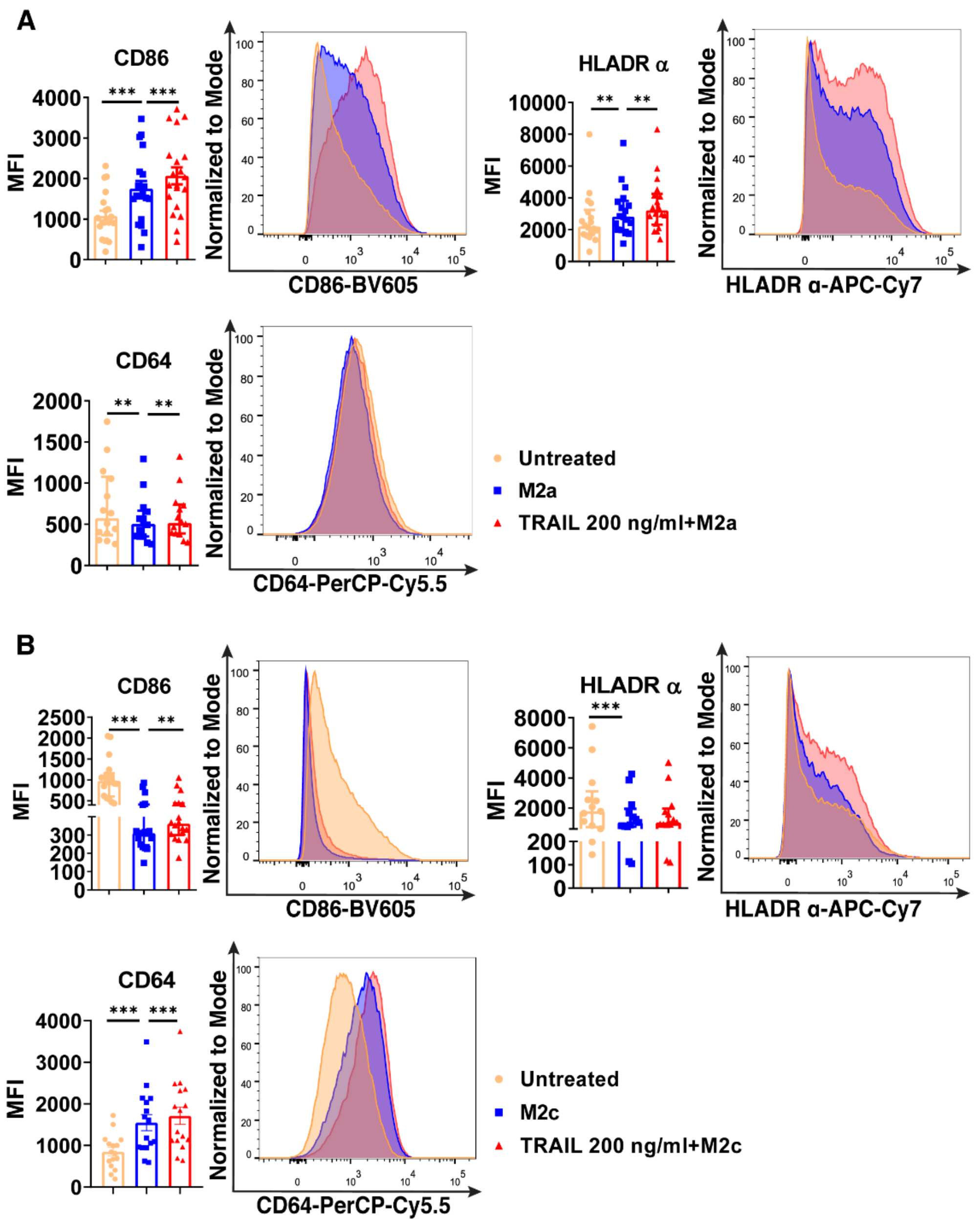

Figure S5. TRAIL increases the expression of classical M1 markers in primary human M2a and M2c macrophage subtypes at the protein level. Macrophages were pre-stimulated with 200 ng/ml TRAIL for 6 hours and then polarized into M2a with 20 ng/ml IL-4 or M2c with 10 ng/ml IL-10 for 12 hours. Control groups were left unstimulated or stimulated with only M2a or M2c polarization factors for 12 hours. Expression of cell surface M1 markers was analyzed by flow cytometry in (A) M2a and (B) M2c macrophages and representative plots are included. Data shown are mean ± SEM or median with interquartile range pooled from four or more independent experiments [(A) n=14-20, (B) n=13-18]. Statistical analyses were performed with a One-way ANOVA with Sidak's multiple comparisons post-hoc test, or Friedman with Dunn's multiple comparisons post-hoc test between untreated and M2 macrophages, M2 and TRAIL-treated M2 macrophages, *P<0.05, **P<0.01, ***P<0.001 **(Supplement of Figure 8)**.

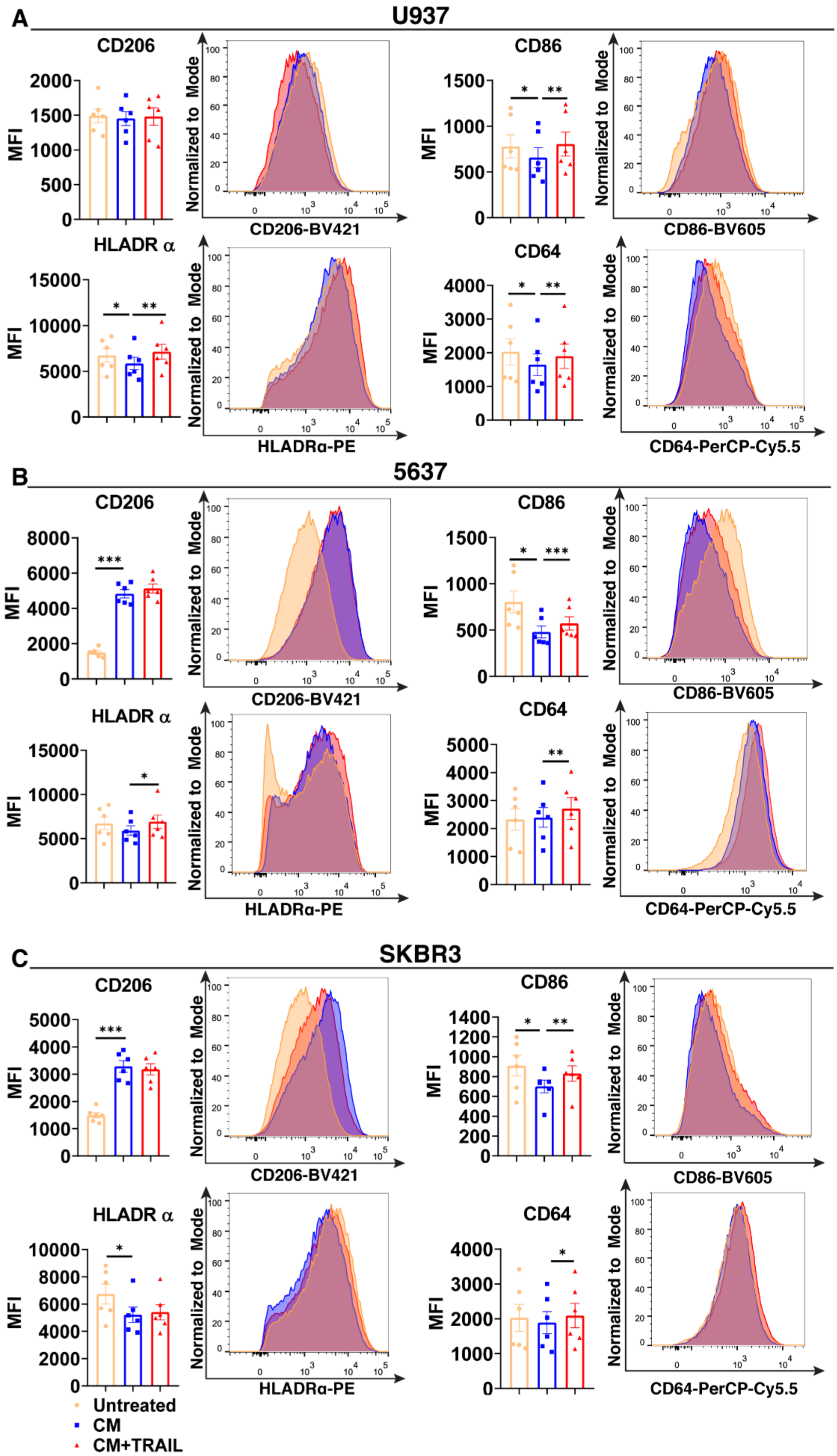

Figure S6. TRAIL increases the expression of M1 markers in tumor-associated macrophages (TAMs). Macrophages were polarized into TAMs with conditioned media (CM) of (A) U937, (B) 5637, and (C) SKBR3 tumor cell lines for 72 hours. TAMs were stimulated

with 200 ng/ml TRAIL in the last 18 hours of incubation without removing the tumor CM. Control group macrophages were left unstimulated or only treated with tumor CM for 72 hours. Expression of CD206 M2 marker and CD86, HLADRα and CD64 M1 markers was analyzed by flow cytometry and representative plots are included. Data shown are mean ± SEM pooled from two independent experiments (n=6). Statistical analyses were performed with a One-way ANOVA with Sidak's multiple comparisons post-hoc test between untreated macrophages and tumor CM treated macrophages (TAMs), TAMs and TRAIL treated-TAMs, *P<0.05, **P<0.01, ***P<0.001.

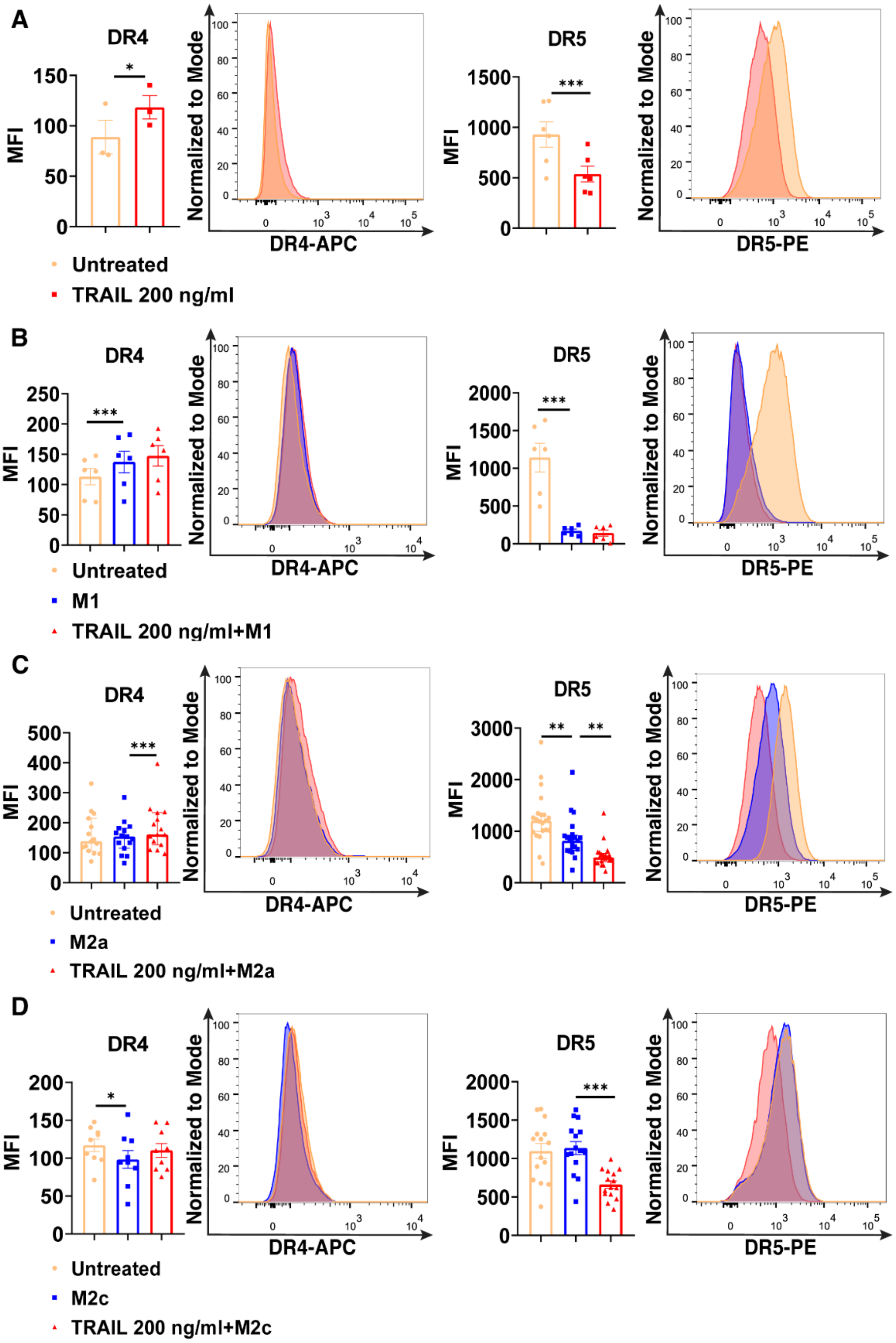

Figure S7. TRAIL increases DR4 and decreases DR5 expression at the cell surface in primary human macrophage subtypes. Macrophages were stimulated with 200 ng/ml TRAIL for 18 hours or pre-stimulated with TRAIL for 6 hours and then polarized into M1 with 100 ng/ml LPS and 20 ng/ml IFNγ, M2a with 20 ng/ml IL-4, or M2c with 10 ng/ml IL-10 for 12 hours. Control groups were left unstimulated or stimulated with only M1, M2a, or M2c polarization factors for 12 hours. Expression of DR4 and DR5 in (A) M0, (B) M1, (C) M2a, (D), and M2c macrophages was analyzed by flow cytometry, and representative plots are included. Data shown are mean ± SEM or median with interquartile range pooled from three or more experiments [(A) n=3-6, (B) n=6, (C) n=15-21, (D) n=9-15]. Statistical analyses were

performed with a two-tailed paired Student's t-test, One-way ANOVA with Sidak's multiple comparisons post-hoc test, or Friedman with Dunn's multiple comparisons post-hoc test between untreated and polarized macrophages, untreated/polarized and TRAIL-treated macrophages, *P<0.05, ***P<0.001.

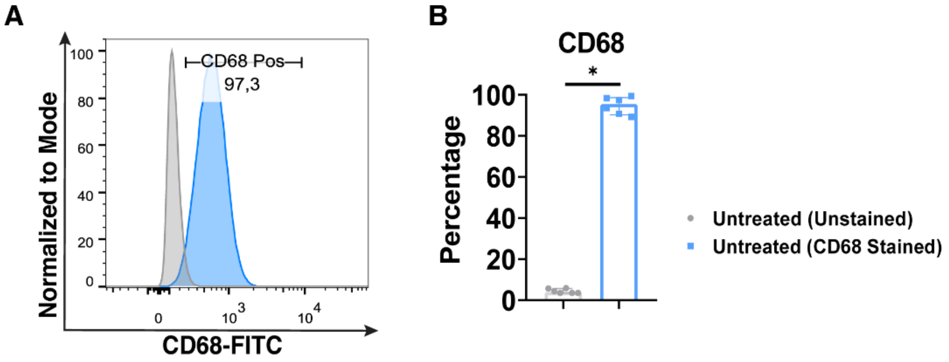

Figure S8. Primary human monocytes are successfully differentiated into macrophages *in vitro*. Monocytes isolated from PBMCs were differentiated into macrophages by incubating the cells in 10 ng/ml M-CSF containing R5 media for 7 days. Expression of human macrophage maturation marker CD68 in M0 macrophages was analyzed by flow cytometry. (A) Representative plot and (B) Bar graph show the percentage of $CD68^+$ cells. Data shown are median with interquartile range pooled from three independent experiments (n=6). Statistical analyses were performed with a Wilcoxon matched-pairs signed-rank test, between unstained and CD68 stained groups, *P<0.05.

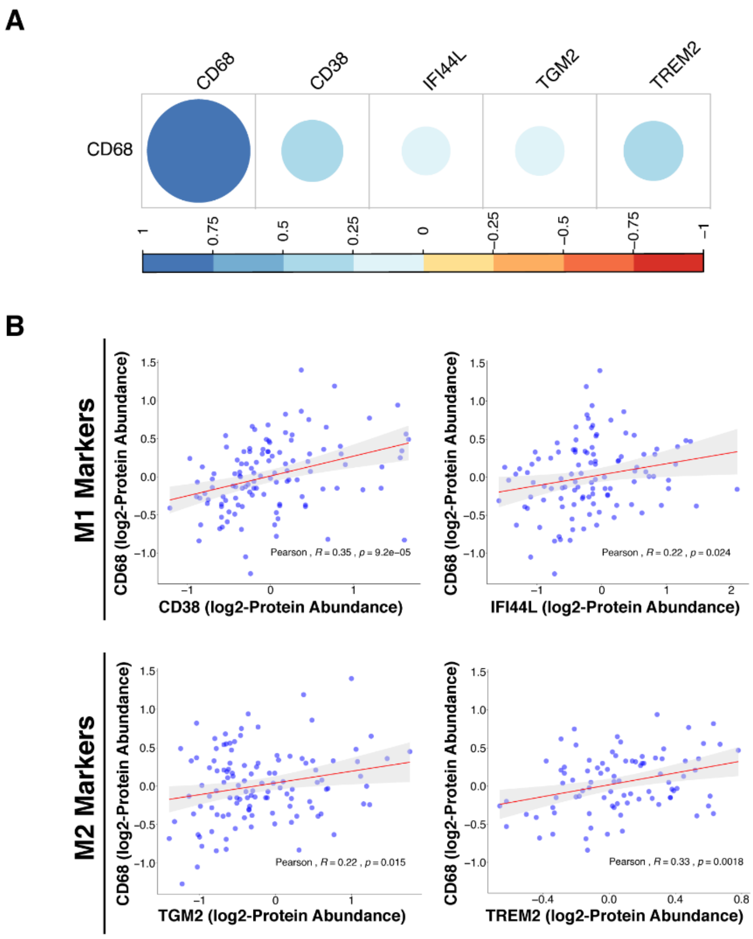

Figure S9. CD68 expression is positively correlated with the expression of selected M1 and M2 markers in ovarian cancer patients. (A) The correlation matrix between the expression of CD68 and M1 (CD38 and IFI44L) or M2 (TGM2 and TREM2) markers in ovarian cancer patients was shown based on the correlation coeffient value. (B) Scatter plots showing the correlation between the expression of CD68 and each M1 or M2 marker were demonstrated with Pearson correlation coefficient and p-values. A positive correlation (R>0) indicates that higher expression of CD68 is related with higher expression of the M1 and M2 markers at the protein level **(Supplement of Figure 14).**
